# The effects of anti–TNF agents on the expansion of T helper-type 17 cells driven by lipopolysaccharide-stimulated monocytes

**DOI:** 10.1186/1479-5876-10-S3-P42

**Published:** 2012-11-28

**Authors:** Gianluca Fossati, Louise Healy, Andrew Nesbitt

**Affiliations:** 1 UCB Pharma, Slough, UK

## Introduction

T helper-type 17 (Th17) cells are proinflammatory CD4+ cells characterized by Interleukin-17 (IL-17) production. Evidence suggests cytokines produce by Th17s, including IL-17, are involved in rheumatoid arthritis (RA) pathogenesis [1]. Lipopolysaccharide (LPS)-stimulated monocytes promote CD4+ cell differentiation into Th17 cells, producing IL-17 *in vitro*[2].

## Aim

Examine the effect of 4 anti–tumor necrosis factor (TNF) agents (adalimumab, etanercept, infliximab, and certolizumab pegol) on CD4+CD45RO+ memory T cell expansion into Th17 cells, driven by LPS-stimulated monocytes.

## Patients and methods

Monocytes and CD4+ cells were purified, by positive and negative selection, from peripheral blood mononuclear cells of healthy volunteers. CD4+CD45RO+ memory T cells were enriched from the CD4+ cell fraction by positive selection. A 1:1 ratio of monocytes and memory T cells was co-cultured for 7 days with CD3/CD28 Human T-Activator Dynabeads and 1 μg/mL LPS. Cells were cultured with and without 10 μg/mL anti-TNF agent. Subsequently, CD4+ cells were stained for intracellular Interferon γ (INFγ) and IL-17A, and analyzed by flow cytometry. IL-17A and IL-17F secretion was determined by ELISA.

## Results

IL-17A-producing CD4+ cell were 2.5-fold less frequent in co-cultures with the 4 anti-TNF agents compared to controls. IL-17A and IFNγ producing CD4+ cell levels were similar between the 4 anti-TNF agents. Compared to controls, IL-17A and IL-17F secretion into the supernatant was lower in anti-TNF exposed co-cultures (580 pg/mL vs. 180 pg/mL and 8 ng/mL vs. 2 ng/mL, respectively). There were no significant differences in the IL-17A or IL-17F concentration between co-cultures exposed to different anti-TNF agents.

## Conclusion

Exposure to anti-TNF agents inhibited Th17 expansion and IL-17A production, suggesting that anti-TNF agents may reduce Th17 expansion and, as a consequence, IL-17A and IL-17F concentration.
